# Predictive models of influenza A virus lethal disease yield insights from ferret respiratory tract and brain tissues

**DOI:** 10.1038/s41598-025-09154-0

**Published:** 2025-07-08

**Authors:** Troy J. Kieran, Xiangjie Sun, Taronna R. Maines, Jessica A. Belser

**Affiliations:** Influenza Division, Centers for Disease Control and Prevention, 1600 Clifton Rd NE, Atlanta, GA USA

**Keywords:** Viral pathogenesis, Influenza virus

## Abstract

Collection of systemic tissues from influenza A virus (IAV)-infected ferrets at a fixed timepoint post-inoculation represents a frequent component of risk assessment activities to assess the capacity of IAV to replicate systemically. However, few studies have evaluated how the frequency and magnitude of IAV replication at discrete tissues contribute to within-host phenotypic outcomes, limiting our ability to fully contextualize results from scheduled necropsy into risk assessment settings. Employing aggregated data from ferrets inoculated with > 100 unique IAV (both human- and avian-origin viruses, spanning H1, H2, H3, H5, H7, and H9 subtypes), we examined relationships between infectious virus detection in four discrete tissue types (nasal turbinate, lung, brain, and olfactory bulb [BnOB]) to clinical outcomes of IAV-inoculated ferrets, and the utility of including these discrete tissue data as features in machine learning (ML) models. We found that addition of viral tissue titer data maintained high performance metrics of a predictive lethality classification ML model with or without inclusion of serially-collected virological and clinical data. Interestingly, infectious virus in BnOB was detected at higher frequency and magnitude among IAV associated with high pathogenicity phenotypes in ferrets, more so than tissues from the respiratory tract; in agreement, BnOB was the highest relative ranked individual tissue specimen in predictive classification models. This study highlights the potential role of BnOB viral titers in assessing IAV pathogenicity in ferrets, and highlights the role ML approaches can contribute towards understanding the predictive benefit of in vivo-generated data in the context of pandemic risk assessment.

**Supplementary Information:**

The online version contains supplementary material available at 10.1038/s41598-025-09154-0.

## Introduction

While primarily considered a respiratory pathogen, influenza A viruses (IAV) are capable of extrapulmonary spread, and a wide range of systemic complications have been reported following human infection with IAV of both human and zoonotic origin^1^. Ferrets inoculated with IAV can recapitulate numerous clinical signs indicative of both respiratory and extrapulmonary disease as observed in humans (including neurological, ocular, and gastrointestinal involvement), making this model well-suited to study IAV pathogenicity^2^. As such, risk assessment activities employing the ferret model typically include collection of systemic tissues from virus-inoculated animals at a fixed timepoint post-inoculation (p.i.) to investigate the incidence, frequency, and magnitude of IAV replication outside of the upper respiratory tract^3^. A recent investigation by our group found that viral titers in the lower respiratory tract were not a principal stand-alone driver of lethal outcomes in ferrets^4^ but extrapulmonary tissues were not assessed. Detection of infectious virus, viral antigen, and/or histological lesions in the central nervous system, brain (cerebrum), and olfactory bulb tissues is often associated with enhanced morbidity and mortality outcomes in IAV-inoculated ferrets^5,6 ^highlighting the necessity of including these specimen types when investigating extrapulmonary spread. However, studies assessing molecular determinants of IAV neurotropism in ferrets are often conducted with limited panels of isogenic viruses^6–9 ^making it challenging to extrapolate findings and assess the relative contribution of specific features to this multifactorial trait to heterogeneous virus populations.

Incorporating machine learning (ML) approaches in risk assessment activities can offer added value and high utility for predicting different phenotypic outcomes in mammals^10,11^. However, these models are only infrequently informed by in vivo-generated data^12,13 ^due in part to the challenges of generating sufficient datasets for model testing/training and complexities in compiling and structuring data from in vivo experimentation into a tidy data analysis-friendly format. Beyond the predictive utility conferred by high-performing ML models, investigation of relative ranked importance of contributing model features can provide valuable insight into biologically meaningful data trends which may otherwise be obscured or overlooked^13,14^. Inclusion of in vivo-generated data as features in predictive models thus represents a needed but understudied area of investigation. Previous work from our group found that viral titer data from ferret nasal wash specimens represented a highly ranked feature across multiple ML classification models assessing lethality and morbidity^13 ^but the relative contribution of respiratory tract and extrapulmonary tissues in these predictive models was not assessed.

Here, we performed exploratory analyses to assess the frequency and magnitude of IAV detection in both respiratory tract (nasal turbinates, lung) and extrapulmonary (brain, olfactory bulb) tissues in ferrets inoculated with a heterogeneous panel of human and zoonotic strains, to determine which molecular and clinical features were statistically linked with detectable virus replication in these tissues on day 3 p.i. To corroborate results, we assessed the relative utility and benefit of including these discrete tissue data into predictive models of ferret lethality and disease severity, to ascertain the relative contribution of IAV replication at different sites to phenotypic outcomes in ferrets. Among all tissues assessed, viral titers in olfactory bulb exhibited the highest statistical linkages with phenotypic outcomes and were the highest ranked individual feature in ML models, highlighting the valuable contribution assessments of IAV replication in this tissue specimen may contribute to risk assessment rubrics.

## Results

### Clinical features associated with virus replication in ferret tissues day 3 p.i.

**Fig. 1:**
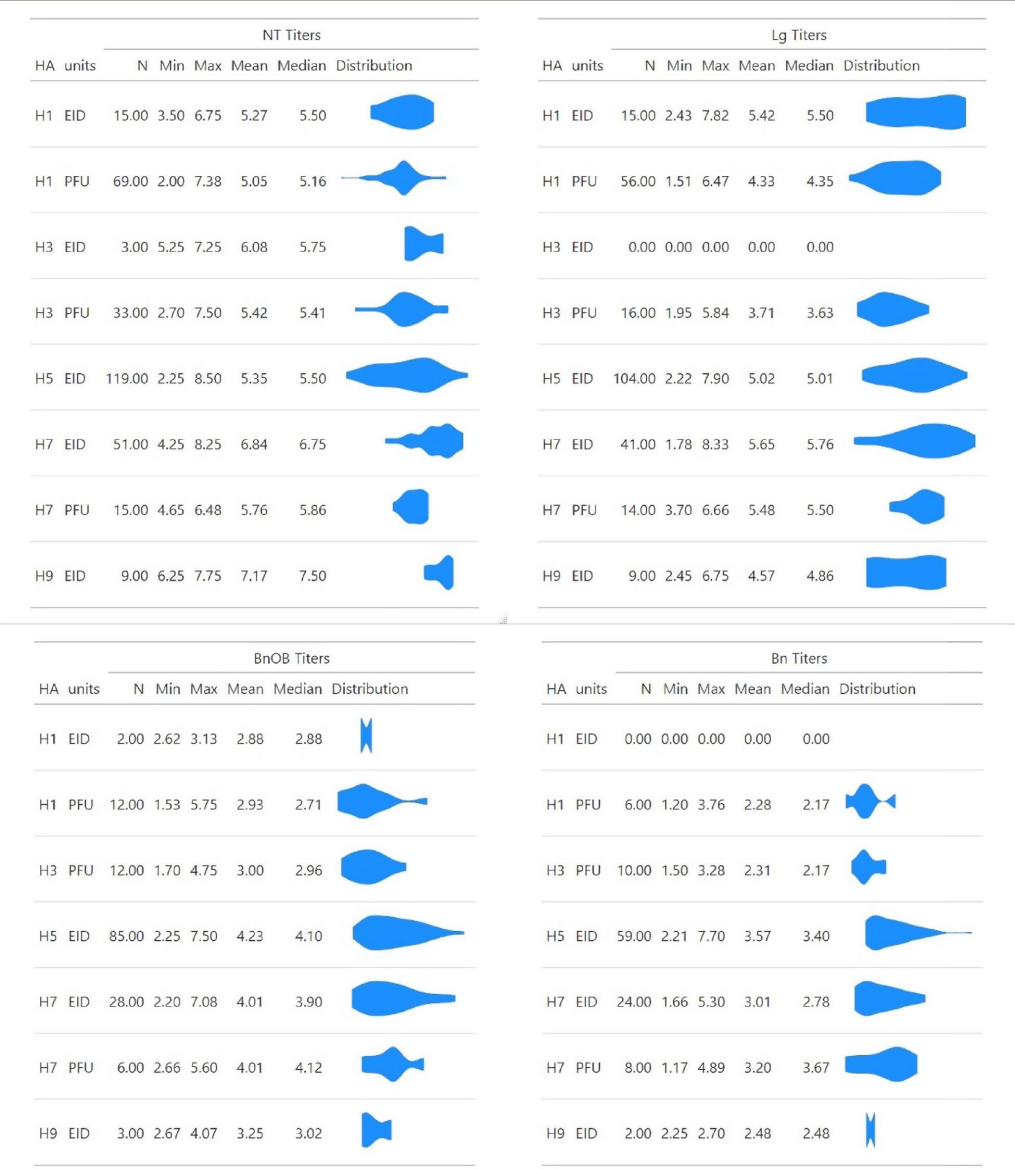
Distribution of viral titers day 3 p.i. in ferret respiratory and extrapulmonary tissues. Infectious virus titers in ferret nasal turbinates (NT), lung (Lg), olfactory bulb (BnOB), and brain (Bn) tissues day 3 p.i. with IAV of different HA subtypes included in the study (sample size for each per-subtype, per-unit group is specified in N). Units denote viruses titrated in eggs (EID) or cells (PFU). Minimum, maximum, mean, and median titers reported as log_10_ infectious units per ml (NT) or gram (all other tissues) are shown alongside a distribution plot.

 Scheduled ferret necropsy at a fixed timepoint (typically day 3 post-inoculation (p.i.) or around the time of peak anticipated virus replication during the acute phase of infection) permits assessment of influenza A virus (IAV) spread throughout the respiratory tract and extrapulmonary sites such as the brain. To explore the capacity of heterogeneous viruses to spread and replicate in systemic mammalian tissues during acute infection, necropsy data from ferrets inoculated with 104 IAV (inclusive of H1, H3, H5, H7, and H9 subtypes) were aggregated from nasal turbinates (NT), lung (Lg), brain (Bn) or olfactory bulb (BnOB) tissue (Supplemental Table 1). Infectious virus from all HA subtypes tested were detected in all tissue types day 3 p.i., though the distribution and magnitude of infectious virus titers when present varied substantially by HA subtype (Fig. 1). We first examined to what extent these viral titers were associated with clinical parameters from ferrets inoculated with homologous virus and observed for 14 days p.i. The relative effect (as captured by the reported p value, with smaller values < 0.05 denoting increasing statistical significance) and magnitude of effect size (as captured by the Cohen’s d standardized mean difference, with a value of 1 indicating the groups differ by one standard deviation and such that higher values confer a larger difference between two means) was determined for each tissue and clinical feature.

In agreement with prior work, elevated viral titers in NT and Lg tissues were detected among ferrets inoculated with viruses associated with enhanced morbidity and mortality metrics, notably per-virus lethal outcomes (Table 1)^4^. However, BnOB viral titers from ferrets inoculated with viruses associated with high lethality and morbidity outcomes (notably ≥ 50% lethality and > 10% weight loss) were associated with the highest effect size (relative to ferrets inoculated with viruses causing low pathogenicity in this species) compared with all other tissue specimens examined, including those from the respiratory tract (Table 1). For all tissues examined, a general trend of higher statistical significance was observed with viruses titered in eggs relative to cells; this is likely because all viruses associated with ≥ 50% lethality, and all IAV classified as HPAI, were egg-titered. These findings support that viruses that cause a high pathogenicity phenotype in ferrets were associated with a higher magnitude of infectious virus detected in both respiratory and brain tissue specimens day 3 p.i., with olfactory bulb tissues displaying the highest statistical significance and relative effect size among all tissues evaluated.

**Table 1:** Magnitude of virus replication in respiratory tract and extrapulmonary tissue specimens collected day 3 p.i.

Tissue^a^	Feature	Variable 1		Variable 2			
		Condition	Mean titer^b^	Condition	Mean titer	p value^c^	Cohen’s d^d^
BnOB	Lethality^e^	≥ 50%	4.93 EID_50_	< 50%	3.70 EID_50_	4.89e-7	0.990872
	Weight loss^e^	> 10%	5.00 EID_50_	< 10%	3.78 EID_50_	2.62e-7	0.990631
	Weight loss	> 10%	3.42 PFU	< 10%	3.16 PFU	0.659331	0.23679
	Temperature	≥ 1 °C	4.24 EID_50_	< 1 °C	3.41 EID_50_	0.003352	0.668587
	Temperature	≥ 1 °C	3.16 PFU	< 1 °C	3.23 PFU	0.92574	-0.05936
Bn	Lethality	≥ 50%	3.66 EID_50_	< 50%	3.21 EID_50_	0.076922	0.402459
	Weight loss	> 10%	3.75 EID_50_	< 10%	3.14 EID_50_	0.013479	0.54364
	Weight loss	> 10%	2.11 PFU	< 10%	2.67 PFU	0.025558	-0.54928
	Temperature	≥ 1 °C	3.49 EID_50_	< 1 °C	2.41 EID_50_	0.001969	0.957759
	Temperature	≥ 1 °C	2.65 PFU	< 1 °C	2.34 PFU	0.616514	0.311542
NT	Lethality	≥ 50%	6.52 EID_50_	< 50%	5.59 EID_50_	9.11e-6	0.634403
	Weight loss^e^	> 10%	6.51 EID_50_	< 10%	5.62 EID_50_	0.000118	0.606623
	Weight loss	> 10%	5.15 PFU	< 10%	5.26 PFU	0.629701	-0.12554
	Temperature	≥ 1 °C	5.93 EID_50_	< 1 °C	5.30 EID_50_	0.01595	0.43149
	Temperature	≥ 1 °C	5.28 PFU	< 1 °C	5.11 PFU	0.450098	0.187857
Lg	Lethality	≥ 50%	5.86 EID_50_	< 50%	4.93 EID_50_	8.81e-5	0.596728
	Weight loss^e^	> 10%	6.03 EID_50_	< 10%	4.92 EID_50_	2.31e-5	0.711069
	Weight loss	> 10%	4.15 PFU	< 10%	4.45 PFU	0.538817	-0.22164
	Temperature	≥ 1 °C	5.30 EID_50_	< 1 °C	4.64 EID_50_	0.025662	0.416241
	Temperature	≥ 1 °C	4.35 PFU	< 1 °C	4.55 PFU	0.482748	-0.16211

^a^BnOB, olfactory bulb, 148/320 individual ferrets with positive virus detection. Bn, brain, 109/326 individual ferrets with positive virus detection. NT, nasal turbinate, all individual ferrets tested (*n* = 326) with positive virus detection. Lg, lung, 264/326 individual ferrets with positive virus detection. ^b^Mean titer reported among ferrets with positive virus detection either titered in cells (PFU) or eggs (EID_50_), reported as log_10_ titer/g or ml. Limit of detection was 1 log_10_ PFU or 1.5 log_10_ EID_50_. ^c^p value calculated from t test. ^d^Cohen’s d calculated with Hedges’ Correction and assuming unequal variances. ^e^Lethality defined as ≥ or < 50% mortality on a per-virus basis. Weight loss as defined as > or < 10% maximum weight loss on a per-ferret basis between days 1–14 p.i. Temperature as defined as ≥ 1 °C maximum rise on a per-ferret basis between days 1 and 5 p.i.

### Benefit of day 3 p.i. tissue inclusion in supervised classification models of lethality and morbidity

**Fig. 2:**
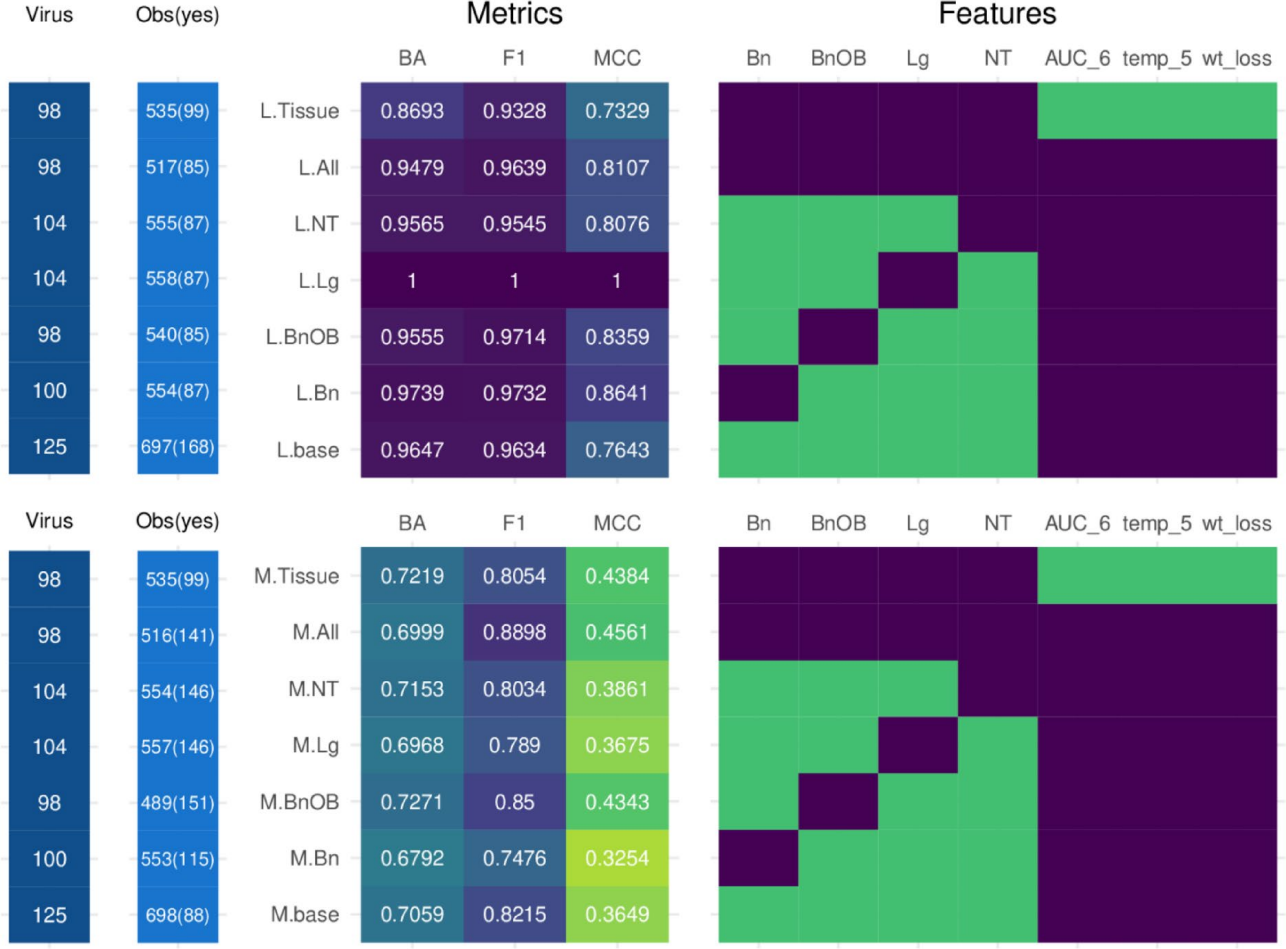
Comparison of lethality and morbidity model performance metrics and feature selection iterations. The number of unique total viruses in the testing and training datasets (Virus), and binary observations based on the classification model tested (Obs(yes)), for each model iteration shown. Heat map depicting balanced accuracy (BA), F1 score (F1), and Matthew’s Correlation Coefficient (MCC) performance metrics for lethality (L, top) or morbidity (M, bottom) models employing different feature selections (.base = base model; NT/Lg/BnOB/Lg = base model with addition of single tissue specified; All = base model with addition of all four tissue types; Tissue = tissue model). Values range from 0 (worst, green) to 1 (best, purple) for all metrics except MCC, which ranges from − 1 (no agreement) to 1 (agreement). Feature inclusion for all lethality (top) or morbidity (bottom) models shown. Purple, feature inclusion; green, feature exclusion. All L1 models include MBAA, RBS, and PA features (not shown). Individual feature definitions features tested are provided in Supplemental Table 4. Full scope of all model metrics for models are reported in Supplemental Tables 5–6.

 Our group recently evaluated the utility of machine learning (ML) algorithms employing in vivo-generated data to predict different phenotypic outcomes, including lethality and disease severity^13^. Both models considered properties of the virus (presence/absence of a multibasic amino acid HA cleavage site (MBAA), predicted receptor binding specificity (RBS), predicted polymerase activity (PA) and clinical/titer data from inoculated ferrets (area-under-the-curve of nasal wash [NW] titers, peak temperature, peak weight loss [lethality model only]). As day 3 p.i. tissue titers from the respiratory tract were statistically associated with several clinical parameters in IAV-inoculated ferrets (Table 1), we next investigated if inclusion of tissue titer data (and not just NW specimen data) could maintain and/or improve predictive models employing serially-collected titer and clinical data from this model. Mean day 3 p.i. viral titers for each tissue were linked with per-ferret records; the number of unique total viruses in the testing and training datasets, and binary observations based on the classification model tested, are specified for each model iteration in Fig. 2.

Building upon our previously established lethality model (L.base, which predicts ferret mortality for any cause between days 1–14 p.i. as a yes/no)^13^, average day 3 p.i. tissue titers were added to L.base individually (L.Bn, L.BnOB, L.NT, L.Lg) or collectively (L.All) (Supplemental Tables 2–3). To compare relative model performance, we employed a panel of single-value metrics to summarize model performance (Balanced Accuracy (BA), F1 score, and Matthew’s Correlation Coefficient (MCC)) for each model iteration. Balanced Accuracy is the average of sensitivity and specificity for each class, useful for imbalanced data. F1 score balances recall and precision for the false positives and negatives. Matthew’s Correlation Coefficient considers all elements of the confusion matrix (true and false, positive and negative predictions), ideal for binary classification and imbalanced data. BA and F1 range from 0 (completely inaccurate) to 1 (completely accurate) while MCC ranges from − 1 (completely inaccurate) to 1 (completely accurate) with 0 being random prediction. The L.base model was previously shown to be high-performing^13^; addition of tissue titer data individually or collectively maintained the high performance observed in the base model, with only modest tissue-specific modulation of model metrics noted (Fig. 2, Supplemental Tables 2–3). Simplification of the L.All model by removing peak weight loss (wt_loss), peak temperature between days 1–5 p.i. (temp_5), and nasal wash titer data (area-under-the-curve from NW specimens days 1–6 p.i., AUC_6) (L.Tissue) maintained the high performance metrics of L.All, indicating a strong effect of day 3 p.i. viral tissue titers on predictive capabilities in this model, even in the absence of serially-collected viral titer or clinical data.

Employing a similar strategy, we next assessed the relative benefit of day 3 p.i. tissue titer inclusion on an underperforming predictive morbidity model (M.base, which predicts maximum ferret weight loss ≥ 14.5% from preinoculation baseline between days 1–14 p.i. as a yes/no)^13^, comparing the same BA, F1, and MCC metrics (Supplemental Tables 7–8) to the base model (Fig. 2). Similar to the lethality model, only modest changes to performance metrics were noted following inclusion of either single tissues or all tissues collectively to the base model, supporting that inclusion of additional viral titer-based factors preserved model functionality in the absence of substantial benefit. Simplification of the M.All model by removing temperature and AUC nasal wash titers (M.Tissue) yielded similar, or modestly improved, performance metrics as M.base, suggesting that the underperforming nature of this model was due to parameters independent of the features included for training, such as the classification parameters of the outcome variable being predicted. Collectively, these results show that inclusion of day 3 p.i. viral tissue titer data can maintain the performance metrics of a predictive classification model using serially collected virological and clinical data, and may provide a meaningful substitute for serially-collected virological and clinical data, but cannot substantially improve performance metrics of an underperforming predictive model.

### Relative feature importance for day 3 p.i. tissue-containing lethality and morbidity models

Preservation of model performance metrics following the addition of day 3 p.i. tissue titer data (L.All and M.All relative to base models) prompted us to examine the relative ranked importance of respiratory and extrapulmonary day 3 p.i. tissues in both classification models, to better ascertain which tissues (and/or combination of tissues) were responsible for the performance metrics obtained.

**Fig. 3:**
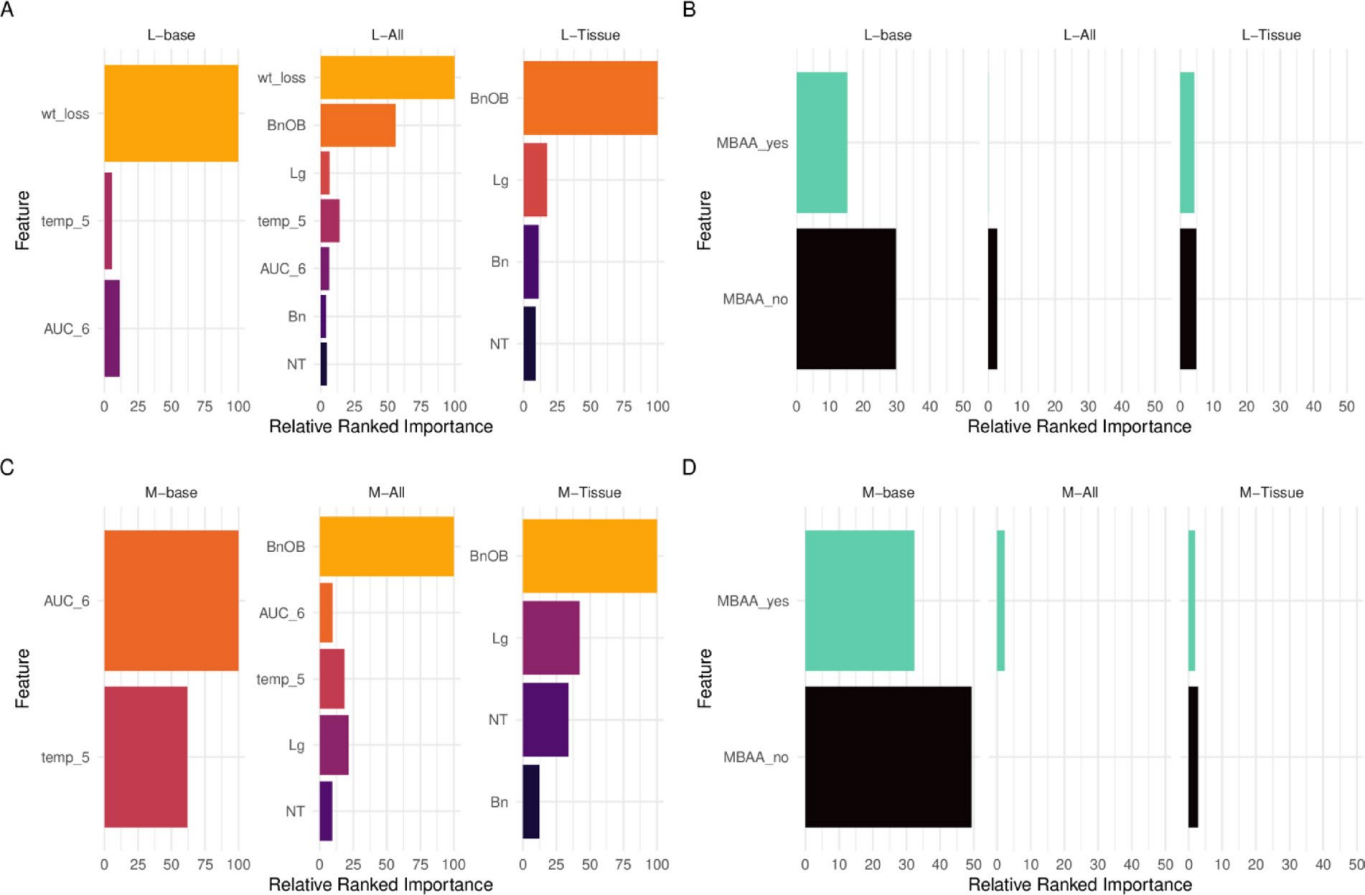
Variability in feature selection among lethality and morbidity models employing different features. Relative ranked importance of top numeric features included in lethality (**A**) and morbidity (**C**) models. Relative ranked importance of the presence (yes) or absence (no) of a MBAA HA cleavage site in lethality (**B**) or morbidity (**D**) models. Relative ranked performance among features of all models is shown in Supplemental Tables 9, 11. Relative ranked importance values are set to 100 for the most important feature and scaled to relative importance for remaining features independently within each model. For each model features are consistently scaled but separated out for visual purposes, and not meant to convey direct feature comparisons, only relative ranking.

Weight loss was previously shown to be the most important predictive variable in the L.base model^13^; it remained the top variable even following addition of all four tissues (L.All). Interestingly, BnOB was identified as the highest ranked importance tissue in this combined model, ranking higher than all other individual tissue types (NT, Lg, and Bn) or serially-collected virological or clinical data included (Fig. 3A, Supplemental Table 9). The high ranked importance of BnOB was maintained in the L.Tissue model that removed serially-collected data, showing the overall importance of BnOB as a predictive measure in these models. This was also the case for molecular variables such as MBAA which has relatively high importance in L.base, higher than AUC and temperature, but was drastically reduced in importance when tissues were added to the models (L.All, L.Tissue) (Fig. 3B). Predictive Power Scores (PPS, a normalized metric, like correlation coefficients, that measures the strength of relationship between variables in both linear and non-linear relationships) supported this pattern with tissues being more or about equal to weight loss in predictive capability (Supplemental Table 10).

For the morbidity base model (M.base), AUC of NW titers and temperature were the two most important variables^13^. Inclusion of all four tissue types once again identified BnOB as the most important tissue feature relative to NT, Lg, and Bn; of note, BnOB displaced AUC_6 (an area-under-the-curve summary metric of serially-collected NW specimens collected between days 1–6 p.i^4^) as the highest relative ranked feature in this model (Fig. 3C, Supplemental Table 11). Similar to what was observed with the lethality model, removal of serially-collected virological and clinical data in the M.Tissue model preserved BnOB as the tissue type with the highest relative ranked importance. For molecular based variables, such as the presence of a multibasic amino acid HA cleavage site (MBAA) which is relatively important in M.base, the importance of this feature was diminished to negligible when tissues were added (M.All, M.Tissue) (Fig. 3D). PPS supported the predictive capabilities of tissue titers (Supplemental Table 12). These analyses support the valuable role of BnOB tissue collection and highlights the predictive utility of this tissue type in the context of pandemic risk assessment and generation of phenotypic classification models.

### Interrelatedness of infectious IAV detection in Bn and BnOB tissue day 3 p.i.

**Fig. 4:**
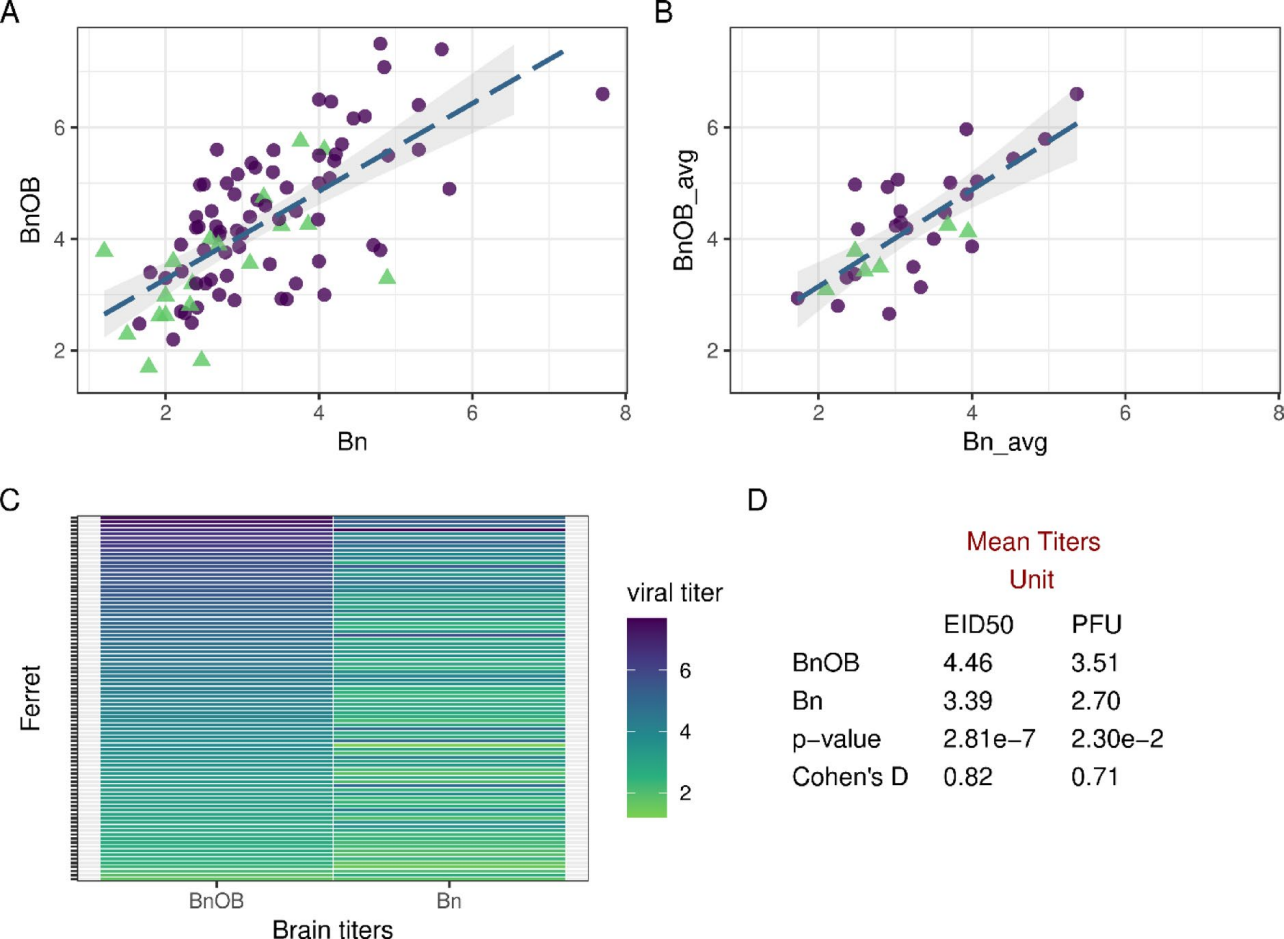
Correlation between ferret brain and olfactory bulb viral titers day 3 p.i. Day 3 p.i. brain (Bn) vs. olfactory bulb (BnOB) titers reported for all ferrets with detectable infectious virus in both tissue types (**A**) or summarized as a per-virus mean when > 50% of inoculated ferrets possessed detectable infectious virus in both tissue types (**B**). Viral titers correspond to the log_10_ of the viral titers, measured in EID_50_/mL (viral titration in eggs, purple circles) or PFU/mL (viral titration in cells, green triangles) on a per-virus basis. Best-fit line determined by linear regression (shading indicates standard error). Titration matrix for all viruses is specified in Supplemental Table 1. Heatmap (**C**) of the per ferret brain viral titers between BnOB and Bn. Overall mean titers (**D**) by titration matrix with p-value from a t test comparing means, and Cohen’s D effect sizes of differences between tissues.

 The highly ranked importance of BnOB (but not Bn) tissue in ML predictive models prompted us to more closely examine features associated with detectable virus replication in both tissues. The anatomical proximity between brain and olfactory bulb tissue would suggest that the incidence and magnitude of viral detection in these tissues is linked, but a close examination of linear correlations between Bn and BnOB specimens has not been previously conducted. When infectious virus was detected in both tissues from an individual ferret, there was a high linear correlation detected between Bn and BnOB (Pearson correlation coefficient [95% CI] *r* = 0.68 [0.55, 0.78], RS-*p* = 1.89e-13) (Fig. 4A, Supplemental Table 13). This association was maintained when employing mean titers for viruses that replicated to detectable titer in a majority of sampled animals in both tissues (*r* = 0.75 [0.54, 0.87], RS-*p* = 1.24e-6) (Fig. 4B), or when viruses were stratified by host origin, predicted receptor binding specificity, or HPAI designation (Supplemental Table 13). In ferrets where infectious virus was recovered from both specimens, viral titers from BnOB were detected at higher mean titer than the paired Bn specimen (Fig. 4C-D). However, there were instances where infectious virus was detected in one tissue and not the other, likely reflective of independent introductions of virus to each tissue, artifacts of sampling a larger tissue, and other reasons; BnOB viral detection in the absence of Bn detection was the more frequent event. Interestingly, viral titers in both Bn and BnOB tissues were capable of reaching high titers (> 10^5^ infectious units) without detected spread in the other, supporting that viral compartmentalization can take place despite close anatomical proximity.

To further elucidate the role anatomical proximity and high viral replication may contribute to incidence and magnitude of virus detection in Bn and BnOB specimens, we assessed the linear correlation between both tissue types with another proximal specimen, nasal turbinate (NT) tissue. Correlations were low (BnOB, *r* = 0.27 [0.11, 0.41], RS-*p* = 9.5e-4) or absent (Bn, *r*= -0.01 [-0.20, 0.18], RS-*p* = 0.93) against NT; generally comparable results were present when employing mean titers for viruses with > 50% virus detection and not per-ferret values (Supplemental Table 13). Linear correlations remained low (*r* ≤ 0.35) between BnOB and NT tissue titers when viruses were stratified by host origin, predicted receptor binding specificity, or HPAI designation (Supplemental Table 13). These findings support that the high correlation noted between viral titers detected in BnOB and Bn tissue is not primarily attributable to the magnitude of viral titers at other proximal tissues such as NT.

### Virological features associated with virus detection in Bn and BnOB tissues day 3 p.i.

 While infectious virus is typically detected in certain upper respiratory tract specimens (e.g. NW, NT) among all ferrets following high-tier virus inoculation^4 ^viral detection in the olfactory bulb and/or brain can range from 0 to 100% of inoculated animals depending on the strain used to infect. In this study, 49.5% and 35% of all tested IAV were detected at > 50% frequency in BnOB and Bn tissues, respectively (Table 1). We employed Fisher’s Exact tests to identify virological features statistically associated with detection of a given virus among > 50% of collected specimens, to ascertain the extent to which the higher predictive utility of BnOB tissue titers relative to Bn could be attributed to virological correlates of replication. Notably, IAV meeting the classification of HPAI were 29.5 times more likely to be associated with > 50% detection in BnOB compared with viruses not meeting this classification (Table 2). Other features associated with increased frequencies of IAV detection in BnOB were avian origin (relative to mammalian origin viruses), or viruses with any predicted binding capacity to α2,3 linked sialic acids (relative to viruses associated with predicted binding capacity to only α2,6 linked sialic acids) (Table 2). These features were also associated with increased frequency of virus detection in Bn tissue, albeit at lower odds ratios and higher p values than BnOB. In contrast, predicted polymerase activity and the presence of a lysine or glutamic acid at PB2-627 were not found to be consistently associated with higher odds ratios of virus detection in BnOB and Bn tissue. In agreement with findings from ML feature selection, viruses associated with high lethality in ferrets (all-cause lethal events documented in > 50% of ferrets inoculated) were 20.8 times more likely to be associated with > 50% detection in BnOB tissue compared with viruses not meeting this classification, with other clinical parameters not strikingly associated with higher odds ratios of virus detection in either tissue (Table 2).

**Table 2:** Frequency of virus replication in olfactory bulb and brain tissue specimens collected day 3 p.i.

Feature	Variable 1^a^	Variable 2	Olfactory bulb^b^		Brain^c^	
			p value	odds ratio^d^	p value	odds ratio^c^
Origin	Avian	Mammalian	5.308e-7	11.8 (3.81, 44.76)	0.001947	4.92 (1.61, 18.28)
HPAI	Yes	no	3.303e-12	29.52 (8.98, 119.87)	0.0003158	5.02 (1.95, 13.72)
RBS	A	H + D	3.046e-7	9.82 (3.61, 29.32)	0.01077	3.33 (1.27, 9.4)
RBS	A + D	H	6.873e-5	8.45 (2.51, 37.32)	0.01703	3.92 (1.16, 17.2)
PA	A	H	0.008282	3.2 (1.31, 8.06)	0.8395	1.1 (0.46, 2.7)
PB2-627	K	E	0.1913	2.09 (0.65, 7.22)	0.02796	3.3 (1.01, 11.27)
Lethality^e^	High	Low	8.165e-5	20.76 (2.94, 907.49)	0.005109	5.75 (1.49, 27.47)
Weight loss^f^	> 10%	< 10%	0.03101	3.68 (1.01, 16.98)	0.001003	7.45 (2.01, 34.89)
Temp	≥ 1 °C	< 1 °C	0.02298	4.16 (1.09, 23.82)	0.1396	2.31 (0.77, 7.56)

^a^Variable 1 lists the parameter for each Feature indicated associated with a positive odds ratio. ^b^BnOB, olfactory bulb. 148/320 individual ferrets with positive virus detection (46.3%). 49 viruses included in analysis where infectious virus titers were detected in a majority of all tested specimens (49.5% of all viruses tested). ^c^Bn, brain. 109/326 individual ferrets with positive virus detection (33.4%). 36 viruses included in analysis where infectious virus titers were detected in a majority of all tested specimens (35% of all viruses tested). ^d^Odds ratio reflects higher odds for Variable 1 in all instances (95% confidence interval in parentheses). ^e^Lethality as defined as > 50% (high) or < 50% (low) lethality on a per-virus basis. For BnOB, due to all viruses of high lethality replicating in this tissue, an adjustment factor of 1 was added to all groups to conduct the analysis. ^f^Weight loss as defined as > or < 10% mean maximum weight loss on a per-virus basis. Feature abbreviations include HPAI (highly pathogenic avian influenza), RBS (predicted receptor binding specificity), PA (predicted polymerase activity); with variables representing avian (A), human (H), or dual (D, avian and human).

With few exceptions, the presence of virological features associated with higher odds ratios of virus detection in > 50% of specimens collected were also associated with higher mean viral titers in these specimens (Table 3), though the relative effect (p value) and magnitude of effect size (Cohen’s d) varied by tissue, feature, and titration method employed. Collectively, these findings support that viruses of avian origin and/or bearing hallmarks of avian viruses were statistically more likely to be detected at a high (> 50%) frequency, and higher mean titer when detected, in brain and olfactory bulb tissues of ferrets early (day 3) p.i. relative to IAV bearing features associated with mammalian-adapted IAV, with this effect most pronounced in BnOB tissue relative to Bn.

**Table 3:** Magnitude of virus replication in olfactory bulb and brain tissue specimens collected day 3 p.i.

	Feature	Variable 1		Variable 2		p value^c^	Cohen’s d^d^
		Condition	Mean titer^b^	Condition	Mean titer		
BnOB^a^	Origin	Avian	4.01 PFU	Mammalian	2.96 PFU	0.052684	0.951673
	HPAI	yes	4.21 EID_50_	no	3.50 EID_50_	0.015381	0.579637
	RBS	A	4.20 EID_50_	H + D	3.13 EID_50_	0.003278	0.867334
	RBS	A	4.25 PFU	H + D	3.10 PFU	4.09e-6	1.053815
	PA	A	3.98 EID_50_	H	4.55 EID_50_	0.062702	-0.46014
Bn^a^	Origin	Avian	3.20 PFU	Mammalian	2.30 PFU	0.092558	0.887919
	HPAI	Yes	3.50 EID_50_	No	2.40 EID_50_	0.000519	0.976418
	RBS	A	3.48 EID_50_	H + D	2.03 EID_50_	0.000292	1.271945
	RBS	A	3.67 PFU	H + D	2.50 PFU	0.00658	1.166041
	PA	A	3.34 EID_50_	H	3.51 EID_50_	0.561862	-0.15333

^a^BnOB, olfactory bulb, 148/320 individual ferrets with positive virus detection. Bn, brain, 109/326 individual ferrets with positive virus detection. ^b^Mean titer reported among ferrets with positive virus detection either titered in cells (PFU) or eggs (EID_50_), reported as log_10_ titer/g. Limit of detection was 1 log_10_ PFU or 1.5 log_10_ EID_50_. ^c^p value calculated from t test. ^d^Cohen’s d calculated with Hedges’ Correction and assuming unequal variances. Feature abbreviations include HPAI (highly pathogenic avian influenza), RBS (predicted receptor binding specificity), PA (predicted polymerase activity); with variables representing avian (A), human (H), or dual (D, avian and human).

## Discussion

The capacity for influenza A virus (IAV) spread beyond the mammalian upper respiratory tract represents a complex and multifactorial trait. Despite strong anatomical compartmentalization between distinct sites in the respiratory tract^15,16 ^some IAV are nonetheless capable of spreading to and/or replicating throughout this tissue, even in the absence of initial virus deposition to the lower respiratory tract^17–19^. Beyond respiratory tract tissues, IAV can spread systemically to the central nervous system in ferrets via multiple routes, including the olfactory and cranial nerve, inner ear and vestibulocochlear nerve, and via hematogenous spread^6,20–22^. Given this capacity, respiratory tract and brain specimens are frequently collected during standard risk assessment activities employing novel and emerging IAV. However, studies assessing the relative utility of tissue titers (inclusive of both respiratory and extrapulmonary sites, or viral titers from different areas of the respiratory tract) to inform phenotypic outcomes (notably lethality and disease severity) in ferrets have only infrequently been performed^4,23 ^limiting our ability to fully interpret these data. We employed a combination of standard statistical exploratory analyses and machine learning (ML) techniques to collectively assess the predictive ability these data can contribute to risk assessment activities, identifying an underappreciated role for BnOB viral replication in predicting phenotypic outcomes in IAV-inoculated ferrets.

Among all clinical outcomes examined (lethality, weight loss, temperature rise), lethal outcomes were most strongly linked with infectious IAV detection in discrete tissues day 3 post-inoculation (p.i.) (Table 1). Prior work from our group employing predictive ML classification models supports that all-cause lethal outcomes (driven primarily but not exclusively by virus-infected ferrets reaching humane weight loss cutoffs and/or exhibiting neurological signs) are easier to predict and are more closely linked to specific virological and/or molecular determinants than morbidity (as captured by weight loss), likely attributable to a diverse range of virological and immunological factors (Fig. 2)^13^. As such, the strongest statistical associations (and highest relative effect sizes) observed for viral titers in NT, Lg, and BnOB tissues measured against lethal outcomes, and not weight loss or temperature values, was not surprising. However, the high ranked importance of BnOB tissue titers (relative to NT or Lg tissues), and heightened utility of BnOB compared to Bn tissue data in both statistical and ML settings, was of notable interest. Both seasonal and zoonotic-origin IAV are frequently detected in the lung day 3 p.i., whereas extrapulmonary spread is less frequently observed with low-virulence viruses^24 ^potentially supporting the higher ranked feature importance of BnOB in statistical and ML models relative to the respiratory tract. The higher ranked importance of BnOB relative to Bn could be due to the relatively early (day 3 p.i.) fixed timepoint sampled, which represents acute-phase virus detection; evaluation of tissues collected more proximal to the time of peak morbidity/mortality outcomes (indicative of later-stage pathology and disease progression) could yield different results, and would provide a valuable comparative study to the findings presented here.

Modulating inclusion of day 3 tissue titers in the lethality base (L.base) model revealed an overall trend of improvement (Supplemental Tables 2,3). Each tissue tested, whether included individually or as a group, showed at least some improvement over the base model, with Lg, BnOB, and Bn showing the highest model performance. When serially-collected clinical and viral features were removed from the model, the inclusion of all four tissues together (L.Tissue) nonetheless had similar performance to the base model, supporting that this lethality model could (to some extent) employ different representative viral titer metric features to similar effect. Looking at individual tissue models, the greatest performance was found with BnOB only (L.BnOB), while the rest of the individual tissue models performed worse than the base model. This indicates that we can see some improvements with the inclusion of tissue titers in a lethality model with other serially-collected clinical and viral titer data, however with the exception of BnOB or all tissues together, tissue data cannot replace serially-collected clinical and viral titer data in these predictive models. We observed a similar trend with the morbidity models (Supplemental Tables 7, 8) with modest overall improvement in model performance over base (M.base) with the inclusion of any and all tissue titers. However, the addition of tissue titer data did not offer meaningful improvements to the base underperforming morbidity model comparable to the high performance metrics of the lethality models. This indicates that while tissues could replace certain other features to maintain existing performance metrics of M.base, they could not substantially improve the model, and further investigation of features outside the scope assessed here (e.g. non-titer based features like relative levels of proinflammatory cytokine expression in different tissues including the brain^22,25^) is warranted. Furthermore, all features in ML models and statistical analyses were assessed individually; further study assessing linkages between these parameters would also be of benefit.

Numerous studies have assessed the capacity of IAV to spread to the ferret central nervous system (CNS), and infectious virus and viral antigen have been detected in numerous ferret CNS tissues and specimens post-IAV inoculation, including cerebellum, brainstem, spinal cord, and cerebrospinal fluid^5,6,20,22,26^. Furthermore, just as IAV spread throughout the respiratory tract can be modulated by experimental route and dose^27 ^viral invasion of the CNS can be dependent on the experimental conditions employed^28,29^. Extrapulmonary tissues assessed in this study were limited to two representative CNS specimens: the olfactory bulb and the brain (inclusive of caudal and rostral sections) at one fixed timepoint p.i.; infectious virus has been reported in numerous CNS tissues throughout the acute phase of infection with both human and zoonotic IAV^23^. As such, results presented are specific to the conditions employed, and variation of the experimental inoculation methodology (for example, using an intranasal inoculation volume of < 1 ml or conducting aerosol-based inoculation exposures) or CNS sampling used in this work^30 ^could modulate the conclusions drawn here.

In line with prior work, analyses in this study were conducted with both individual per-ferret observations, and per-virus means^4^. When assessing frequency of virus detection in the brain or olfactory bulb, we elected to include viruses for which a majority of inoculated ferrets had infectious virus recovered from a given tissue to differentiate between repeated introductions of virus into a given tissue governed by neurotropism versus stochastic introductions (Table 2). Emergence of *de novo* mutations has been reported in IAV recovered from ferret brain specimens following intranasal inoculation, supporting the capacity for increased fitness of variants following CNS invasion^7^. Our analyses are restricted to viral detection only in the absence of sequencing collected specimens p.i., limiting our ability to determine which stochastic introductions occurring < 50% of the time for a given virus may be attributable to acquisition of mutations facilitating spread into the CNS. The 20 viruses in our dataset with < 50% detection in BnOB encapsulated H1 (pdm09 and variant), H3 (variant), H7 (low pathogenic avian influenza), and H9 subtypes (7 of which had paired Bn titers); the 16 viruses with < 50% detection in Bn were inclusive of H1 (pdm09 and variant), H3 (variant), H5 and H7 viruses (15 of which had paired BnOB titers). These analyses support the capacity for infrequent introductions to brain tissues p.i. with a diversity of viral subtypes (Fig. 1), and indicates the need for enhanced sequencing of viruses when this occurs to investigate commonalities in adaptive mutations that may be present.

Prior studies in vitro and in vivo investigating the molecular determinants governing H5N1 IAV spread to and replication in the brain have identified a role for the multibasic amino acid site (MBAA), and to a lesser extent, a binding preference for α2,3-linked sialic acids in conferring this ability^6,31^. Working with a dataset of viruses including both H5 and H7 subtype viruses bearing this molecular signature, our analyses similarly supported a higher odds ratio of elevated frequency and magnitude of detection among viruses possessing a MBAA than viruses lacking this feature in both CNS tissues evaluated (Tables 2 and 3). A predicted avian receptor binding specificity (with or without the predicted dual capacity to bind α2–6 linked sialic acids) was also similarly linked with CNS tissue detection to detectable titer. Interestingly, while mutations in internal genes such as the polymerase have been implicated in heightened IAV neurotropism^7 ^our analyses did not identify polymerase origin as a meaningful determinant, though future study of other molecular determinants governing neurotropism of diverse IAV is warranted. IAV represents just one of many viral pathogens capable of using the olfactory nerve to reach and spread through the CNS^32 ^supporting similar meta-analytic approaches for other pathogens should sufficient in vivo-generated data exist. Of note, the reduced feature importance of MBAA as a ML variable in both lethality and morbidity models when ferret tissue data were included (Fig. 3B and D) supports the potential utility of the MBAA variable as a meaningful contributor in predicting phenotypic outcomes when tissue data is not available for inclusion.

Several limitations and caveats exist herein. Lack of externally published data sources for consistent tissue titers makes validating these model outcomes with an additional dataset difficult. Models in this study were also not hyperparameter tuned (i.e. selecting optimal machine learning algorithm parameters) in order to provide a consistent direct comparison of models with different features. While not a limitation per se, this leaves room for further model improvements than what we show here; it should be noted that hyperparameter tuning of the L.base and M.base models in a prior study did not drastically alter model performance or outcomes relative to untuned models^13^. However, continued efforts towards meaningful improvements of the morbidity model warrant subsequent investigation (such as assessing different metrics for predicting disease severity beyond peak weight loss p.i. as used in this study), and highlights the role viral spread outside of the upper respiratory tract contributes to this multifactorial trait. While machine learning models can often be difficult to interpret on their own, particularly with regards to biological relevance, when paired with more traditional methods we can gain further insights with consistent patterns and trends that emerge from the data. Furthermore, tissue titer data used to train all models represents day 3 p.i. infectious virus detection only, and did not consider other timepoints before or after this time during the entirety of dynamic IAV infection (however, all but the tissue-only models included AUC_6, a parameter encapsulating area under the curve of infectious virus from nasal wash specimens collected between days 1–6 p.i. (Fig. 2)).

Harmonization of statistical and ML approaches collectively permits identification of previously unrecognized correlates of phenotypic outcomes, and support meaningful biological implications of experimental findings. However, these efforts are limited by a paucity of publicly available repositories of aggregated heterogeneous in vivo data suitable for this work^33^; expansion of these datasets to other research groups and data types will greatly enable and enhance this exciting area of ongoing research in the field, including use of infectious titer values as both model features and outcome variables. While detection of infectious IAV in other extrapulmonary tissues (e.g. spleen, kidney, and liver) is only infrequently reported, further investigations including tissues from other organ systems implicated in IAV pathogenicity beyond the brain (such as gastrointestinal or ocular specimens) are warranted to examine the relative utility of other extrapulmonary specimens in predictive models and, more broadly, for a greater understanding of the interconnectedness of viral replication at discrete sites.

## Methods

### Ferret source data

 All experiments and animal manipulations were performed in accordance with relevant guidelines and regulations, were approved by the CDC Institutional Animal Care and Use Committee (IACUC) in an Association for Assessment and Accreditation of Laboratory Animal Care (AAALAC) International-accredited animal facility and are reported in agreement with ARRIVE guidelines^34^. The primary dataset of serially-collected observations collected from IAV-inoculated ferrets employed for this study has been previously described^4,13^ and is publicly available at data.cdc.gov^33^. Experiments were conducted at either ABSL2 or ABSL3 containment, including enhancements as required by the US Department of Agriculture and the Federal Select Agent Program^35^.

Briefly, ferrets were inoculated under anesthesia (by intramuscular administration of 3:1 ketamine/xylazine solution) with a high dose (10^5^-10^7^ infectious units) of virus intranasally in a 1 ml volume; animals were either observed daily for clinical signs of infection (temperature and weight measurements) and collection of nasal wash specimens every-other-day post-inoculation (p.i.), or humanely euthanized at day 3 p.i. (by intracardiac administration of 1:1 pentobarbital sodium/phenytoin sodium solution) for collection of nasal turbinates (NT), lung (Lg), brain (Bn), and/or brain olfactory bulb (BnOB) specimens. All specimens were titered in 10–11 day old embryonated hen’s eggs or MDCK cells to determine 50% Egg Infectious Dose (EID_50_, limit of detection 10^1.5^ EID_50_/ml) or Plaque Forming Units (PFU, limit of detection 10 PFU/ml) titer and are presented and analyzed as log_10_ titer per ml (NT) or gram (Lg, Bn, BnOB) as described^4^ (Supplemental Table 1).

### Statistical and correlation analyses

 All analyses were conducted in R using version 4.2.1^36^. Infectious virus titers are reported either on a per-ferret basis, or as means per-virus (when a majority of ferrets had detectable infectious virus reported in a specific tissue, and calculating the mean among specimens with positive virus detection only). Pearson product-moment correlations were calculated without adjustment for multiple comparisons and interpreted as strong (*r* > 0.7), moderate (0.3–0.7), weak (0.1–0.3) or none (< 0.1). Odds ratios from Fisher’s exact tests were considered strong (> 3), moderate (1.1–3 and 0.5–0.9) or weak (close to 1). Cohen’s d was calculated using DescTools v0.99.47 R package^37^ using Hedges’ correction and assuming unequal variances. Predictive power scores were calculated with ppsr R package^38^. Figures were created in R using the packages tidyverse (version 1.3.2)^39^, ggplot2 (version 3.4.0)^40^, and patchwork (version 1.2.0)^41^. When reporting p values without adjusting for multiple comparisons, we report a ranking statistic (RS-p) to denote the relative strength of comparisons assessed without stating the absolute measure of statistical significance^42^. P values adjusted for multiple comparisons using Holm’s method^43^ were calculated in R using the package correlation (version 0.8.7)^44^.

### Machine learning (ML) model establishment

 Lethality and morbidity models were previously described utilizing a gradient boosting (gbm) machine algorithm^13^. As previously described, model development and analyses were performed in R v4.2.1 using the packages caret v6.0.93^45^, rsample v1.1.1^46^, and fastDummies v1.6.3^47^, with a split dataset of 70% training and 30% testing using 20x repeated cross validation.

### ML inputs, outputs, and feature selection

The lethality model predicts all-cause mortality of an individual ferret between days 1–14 p.i. as a yes or no. The morbidity model predicts reaching a high weight loss (≥ 14.5%) of an individual ferret between days 1–14 p.i. as a yes or no. Lethality models built off the original variables (L.base) multibasic amino acid (MBAA), predicted receptor binding specificity (RBS), predicted polymerase activity (PA), hemagglutinin subtype (HA), peak weight loss over baseline in grams (wt_loss), mean peak temperature in degrees C change from baseline for days 1–5 post-inoculation (temp_5), and area under the curve per ferret for nasal wash titers days 1–6 post-inoculation (AUC_6)^4^; adding NT, Lg, Bn, BnOB tissues individually and/or collectively (L.All model). Tissue titers are on a per-virus mean if > 50% of sampled ferrets had infectious virus titer detected, otherwise the limit of detection value was used. Additionally, tissues models were examined by excluding serially collected variables wt_loss, temp_5, and AUC_6 (L.Tissue). The morbidity models follow this same strategy with the exception of wt_loss not being a feature present in the original model (M.base^13^), or subsequent models tested here.

No models in this study utilized hyperparameter tuning, including the base models (L.base, M.base), as originally performed^13 ^in order to have a more consistent comparison of tissue variable inclusion compared to the base model. As such, model improvements can be made and may adjust outcomes of relative model comparisons. As previously shown^13 ^there is class imbalance in lethal (15% yes, 85% no) and morbidity (25% yes, 75% no) outcomes, however resampling methods (SMOTE^48^ and ROSE^49^) provided no change to model performance. We obtained several model metrics, and focused on balanced accuracy (BA), sensitivity, specificity, F1 score, and Matthew’s Correlation Coefficient (MCC) to assess model performance.

## Electronic supplementary material

Below is the link to the electronic supplementary material.

Supplementary Material 1

Supplementary Material 2

## Data Availability

The primary datasets from IAV-inoculated ferrets employed for this study are available at data.cdc.gov. Serially-collected data is available as “An aggregated dataset of serially collected influenza A virus morbidity and titer measurements from virus-infected ferrets”^50^. Tissue data is available as: “An aggregated dataset of day 3 post-inoculation viral titer measurements from influenza A virus-infected ferret tissues”^51^. R code used for machine learning analyses and figures is available on Github (https://github.com/Troy-Kieran/machine-learning-influenza-lethality-ferret).
