# Supplementary material for: Predictive models of influenza A virus lethal disease yield insights from ferret respiratory tract and brain tissues
Source: Sci Rep. 2025 Jul 8;15:24342. doi: 10.1038/s41598-025-09154-0 (PMC12234895; doi:10.1038/s41598-025-09154-0)
Supplement: Supplementary file 1 — Supplementary Material 1 [file 41598_2025_9154_MOESM1_ESM.docx]

Table S1. Influenza A viruses for which day 3 p.i. tissues were collected used in this study. _rep indicates if >50% of collected tissues had infectious viruses above the limit of detection (yes) or not (no) for a given tissue (NT, Lg, BnOB, Bn); NA indicates no specimen was collected from the tissue specified.

Table S2. Lethality model metrics for test data predictions. Model features and definitions are in Supplemental Table 4.

Table S3. Confusion matrices and Matthew’s Correlation Coefficient (MCC) for Lethality model predictions. Model features and definitions are in Supplemental Table 4.

Table S4. Model names and features included foe each for Lethality (top) and Morbidity (bottom) models. Feature descriptions defined below.

Table S5. Lethality training model metrics. Model features and definitions are in Supplemental Table 4.

Table S6. Morbidity training model metrics. Model features and definitions are in Supplemental Table 4.

Table S7. Morbidity model metrics for test data predictions. Model features and definitions are in Supplemental Table 4.

Table S8. Confusion matrices and Matthew’s Correlation Coefficient (MCC) for Morbidity model predictions. Model features and definitions are in Supplemental Table 4.

Table S9. Lethality model relative ranked importance values. Importance values are scaled within each model with the top-ranking feature set at 100. Model features and definitions are in Supplemental Table 4.

Table S10. Lethality predictive power scores (PPS) for each feature (defined in Supplemental Table 4) predicting the lethal outcome.

Table S11. Morbidity model relative ranked importance values. Importance values are scaled within each model with the top-ranking feature set at 100. Model features and definitions are in Supplemental Table 4.

Table S12. Morbidity predictive power scores (PPS) for each feature (defined in Supplemental Table 4) predicting the high weight loss outcome.

Table S13. Pearson correlations between Bn and BnOB tissues and relevant virological features.
